# Cancer cell line specific co-factors modulate the FOXM1 cistrome

**DOI:** 10.18632/oncotarget.20405

**Published:** 2017-08-24

**Authors:** Yue Wang, Matthew H. Ung, Tian Xia, Wenqing Cheng, Chao Cheng

**Affiliations:** ^1^ School of Electronic Information and Communications, Huazhong University of Science and Technology, Wuhan, Hubei 430074, China; ^2^ Department of Molecular and Systems Biology, Geisel School of Medicine at Dartmouth, Hanover, NH 03755, USA; ^3^ Norris Cotton Cancer Center, Geisel School of Medicine at Dartmouth, Lebanon, NH 03766, USA; ^4^ Department of Biomedical Data Sciences, Geisel School of Medicine at Dartmouth, Lebanon, NH 03766, USA

**Keywords:** transcription factor, ChIP-seq, FOXM1 reprogramming, genomic binding, breast cancer prognosis

## Abstract

ChIP-seq has been commonly applied to identify genomic occupation of transcription factors (TFs) in a context-specific manner. It is generally assumed that a TF should have similar binding patterns in cells from the same or closely related tissues. Surprisingly, this assumption has not been carefully examined. To this end, we systematically compared the genomic binding of the cell cycle regulator FOXM1 in eight cell lines from seven different human tissues at binding signal, peaks and target genes levels. We found that FOXM1 binding in ER-positive breast cancer cell line MCF-7 are distinct comparing to those in not only other non-breast cell lines, but also MDA-MB-231, ER-negative breast cancer cell line. However, binding sites in MDA-MB-231 and non-breast cell lines were highly consistent. The recruitment of estrogen receptor alpha (ERα) caused the unique FOXM1 binding patterns in MCF-7. Moreover, the activity of FOXM1 in MCF-7 reflects the regulatory functions of ERα, while in MDA-MB-231 and non-breast cell lines, FOXM1 activities regulate cell proliferation. Our results suggest that tissue similarity, in some specific contexts, does not hold precedence over TF-cofactors interactions in determining transcriptional states and that the genomic binding of a TF can be dramatically affected by a particular co-factor under certain conditions.

## INTRODUCTION

Transcription factors (TFs) are crucial proteins that mediate gene transcriptional regulation by binding to specific DNA sequences in all living organisms. Several technologies have been developed to investigate the binding of TFs in a high-throughput manner [1]. Among them, chromatin immunoprecipitation followed by microarray hybridization (ChIP-chip) [2] or high-throughput DNA sequencing (ChIP-seq) [3] have become the most widely used methods to detect binding events of individual TFs across the entire genome [1, 4–6]. These methods identify direct and indirect (through interacting with co-factors) binding sites of DNA-associated proteins of interest [4, 7, 8]. Currently, ChIP-seq has become one of the most important technologies used in genomic studies as evidenced by the rapid accumulation of ChIP-seq data.

Previous studies have reported a rapid turnover rate of binding sites of homologous TFs in different species [9–14], suggesting that individual binding sites of a TF are not conserved. Odom *et al.* performed ChIP-chip analysis and found that the binding sites varied extensively between human and mouse even for TFs that are highly conserved during evolution [13]. Borneman *et al.* compared the pseudohyphal regulators STE12 and TEC1 binding sites in three yeast species, *S. cerevisiae*, *S. mikatae*, and *S. bayanus* under pseudohyphal conditions and reached a similar conclusion [11]. Other than these comparative studies by experiments, computational studies based on systematic motif analysis also indicated high turnover rate of TF binding motifs in different organisms [12, 14]. In spite of this, functional conservation has been demonstrated for many TFs even between species that are distantly related [15, 16]. In other words, the homologous TFs participate in the regulation of the same biological process in different species. Interestingly, the functional conservation of them can be attained through regulating different sets of target genes in different species. For example, Tuch *et al.* showed that the target genes of MCM1 have diverged substantially in three related yeast species; however, in all species MCM1 is involved in regulating cell cycle and mating processes [17]. Moreover, motif analyses indicate that the binding motifs associated with a TF is generally conserved across species, presumably due to the selective pressure imposed on its DNA binding domain [7].

On the other hand, the genomic occupancy of a TF in multiple cell types of the same organism shows different degrees of variation. For some TFs, a high degree of shared occupancy between cell types has been observed. Investigation of CTCF binding in 19 human cell lines, for instance, indicates that on average 72% of CTCF sites were shared between any two cell types [18]. Additionally, variable binding has been observed for 64% of CTCF sites which vary in at least one cell type. However, the binding variation for some other TFs are more dramatic. Shira *et al.* compared the REST genomic occupancy in 16 different human cell lines and found that only 7% of binding peaks are shared by all cell lines [19]. According to the unpredictable binding of TFs described above, an interesting question arises: is the genomic occupancy of a TF more similar in more closely related cell types? Intuitively, this should be the case according to general knowledge from transcriptomic and other genomic studies. It has been shown in previous studies that gene expression [20, 21] and DNA methylation [22, 23] levels are highly consistent in cell lines from the same tissue. Moreover, TF binding is largely determined by local chromatin structure (*i.e.*, the accessibility as measured by DNase I hypersensitivity analysis [24, 25]) that is shaped by epigenetic mechanisms such as histone modification [26, 27]. In fact, it has been demonstrated that a combination of these genomic features with motif analysis can predict TF binding sites with fairly high accuracy [28–32]. Furthermore, data generated from the Encyclopedia of DNA Elements (ENCODE) [33] indicate that high similarity of DNase I hypersensitivity regions between cell lines of similar tissue origins [34, 35]. Given these results, we would also expect TF binding profiles be more similar in closely related tissues or cell types.

In this study, we investigate FOXM1 binding in several cell lines and show that overall genetic similarity of cell lines does not hold precedence over context-specific TF-co-factor interactions in determining TF binding profiles. FOXM1, forkhead box protein, is a crucial cell cycle regulator [36, 37], which has been shown to be highly associated with multiple cancer types [38–42]. Recently, genomic binding data of FOXM1 have been generated by ChIP-seq experiments in several studies [43–47]. Based on these data, we performed a comparative analysis to identify the common and specific genome-wide binding events of FOXM1 in 8 distinct cell lines derived from 7 different tissues. By systematically comparing the binding sites and target genes of FOXM1, we find that even though MCF-7 and MDA-MB-231 are both breast cancer cell lines, FOXM1 binding events are substantially different between these two cell lines compared to non-breast cell lines. In particular, FOXM1 binding sites are more similar in MDA-MB-231, HeLa, U2OS, HEK293, GM12878, SK-N-SH and ECC-1 although they all represent different tissues. Moreover, the prognostic value of FOXM1 has been reported in several cancer types [43, 48–50] with the observation that FOXM1 activity is more predictive to prognosis than its mRNA level. We examined the ability of using target genes to infer FOXM1 activity in tumor samples and investigated their association with patient survival in breast cancer. We find that the inferred regulatory activity of FOXM1 is predictive of the survival of patients, and more interestingly, scores inferred based on FOXM1 targets from different cell lines provide complementary clinically related information -- MCF-7 specific FOXM1 targets inform estrogen receptor (ER) activity while targets in other cell lines inform the proliferative ability of tumor cells. These results indicate that the genomic occupation of TFs is more complicated than expected, and that these nuanced changes in binding activity are manifested at the clinical level.

## RESULTS

### Overview of our analyses

**Figure 1:**
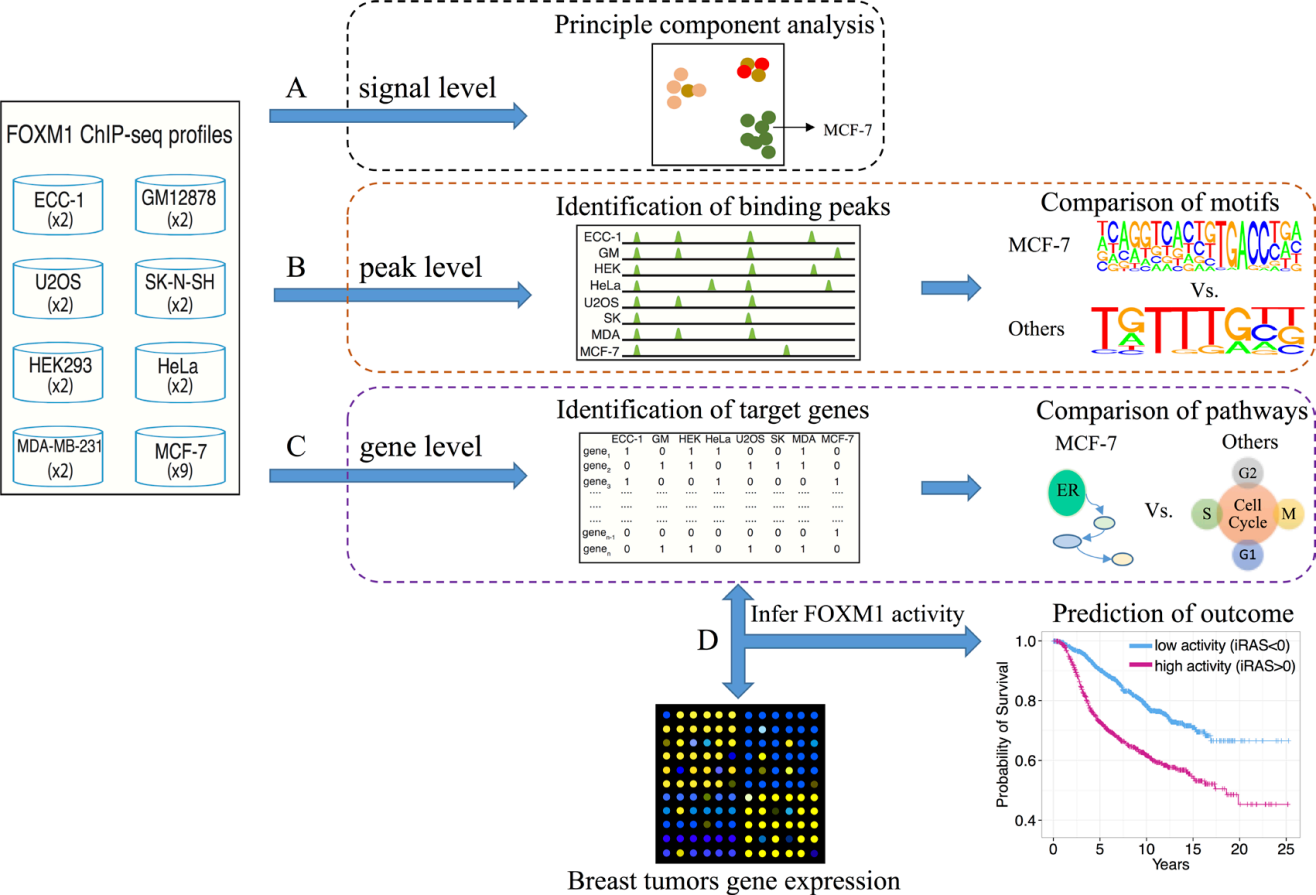
Schematic depicting the comparison of FOXM1 binding in different cell lines We compared the difference based on three levels, (**A**) the raw signal profiles, (**B**) binding peaks and (**C**) target genes, to show the different binding of FOXM1 in different cells. (**D**) We applied the target gene profiles to infer FOXM1 activity and further compared the difference.

To compare FOXM1 binding sites in different human cell lines, we searched for all available FOXM1 ChIP-seq datasets in the Gene Expression Omnibus (GEO) database [51] and obtained a total of 23 ChIP-seq experiments for FOXM1 at the time of writing. These FOXM1 binding data were collected from 6 studies across 8 different cell lines (Table 1), including MCF-7 (ER-positive breast cancer), MDA-MB-231 (ER-negative breast cancer), ECC-1 (endometrium cancer), GM12878 (blood), HEK293 (kidney cancer), HeLa (cervical cancer), SK-N-SH (neuroblastoma), and U2OS (osteosarcoma). For these datasets, we performed systematic comparative analyses using different levels of information from these data (Figure 1). First, at the binding signal level we performed principle component analysis (PCA) [52] on the normalized binding signals of the ChIP-seq experiments. Second, at the peak level we identified FOXM1 binding peaks in each of these ChIP-seq experiments and examined the number of shared peaks between each pair of experiments. Moreover, we performed motif analyses to examine the enrichment of 687 motifs available from the TFANSFAC [53] and JASPAR [54] databases in the binding peaks of each ChIP-seq experiment. Third, at the gene level we defined FOXM1 target genes using a probabilistic model and compared the shared genes between all pairs of experiments. Comparative analyses at the signal, peak, and gene target levels consistently support that MCF-7 ChIP-seq experiments are highly similar to each other but exhibit little resemblance to MDA-MB-231, which is more similar to non-breast cell lines. Finally, at the level of regulation activity, we applied the Binding Association with Sorted Expression (BASE) algorithm [55] to a primary breast cancer expression dataset to infer FOXM1 regulatory activity in patient samples based on its target gene expression. Our results suggested that FOXM1 target genes identified in all cell lines except MCF-7 are informative of the proliferation-regulating activity of FOXM1, while the target genes identified in MCF-7 cell line reflect ER activity rather than FOXM1 activity. Excitingly, the activities inferred based on MCF-7 and other cell lines can be combined to achieve more accurate prediction of patient prognosis.

**Table 1:** Numbers of reads, peaks and genes in each ChIP-seq experiment

ChIP-seq ID	Total Reads	Total Peaks	Total Target Genes	GSE	Antibody	Cell Line	Tissue
SRR577922	116,488,388	18,765	274	GSE32465	SC-502	ECC-1	Endometrium
SRR577923	94,505,444	5,892	228				
SRR577673	168,945,472	54,916	243	GSE32465	SC-502	GM12878	Blood
SRR577674	121,585,588	30,273	199				
SRR1534936	140,964,036	3,883	162	GSE60032	GTX-102170	HEK293	Kidney
SRR1534937	144,836,032	1,963	148				
SRR1045855	68,473,196	3,789	243	GSE52098	SC-502	HeLa	Cervical
SRR1045856	67,826,668	1,426	209				
SRR2390493	168,903,060	12,214	112	GSE72977	SC-501	MCF-7	Breast
SRR2390494	143,504,268	5,032	97				
SRR2390496	150,878,452	14,806	95				
SRR567275	125,468,568	34,091	114	GSE40762	SC-502		
SRR567277	115,745,436	2,763	92				
SRR567284	54,319,080	517	95				
SRR567287	135,548,148	9,148	109				
SRR577748	140,926,888	24,926	95	GSE32465	SC-502		
SRR577749	67,248,588	7,801	90				
SRR567279	112,861,044	13,816	190	GSE40762	SC-502	MDA-MB-231	
SRR567281	129,971,924	11,160	111				
SRR577710	119,592,208	13,405	170	GSE32465	SC-502	SK-N-SH	Neuroblastoma
SRR577711	94,138,820	12,122	183				
SRR500261	147,600,792	1,188	127	GSE38170	SC-502	U2OS	Osteosarcoma
SRR500262	45,863,008	584	138				

### Comparison of FOXM1 binding signals and peaks

**Figure 2:**
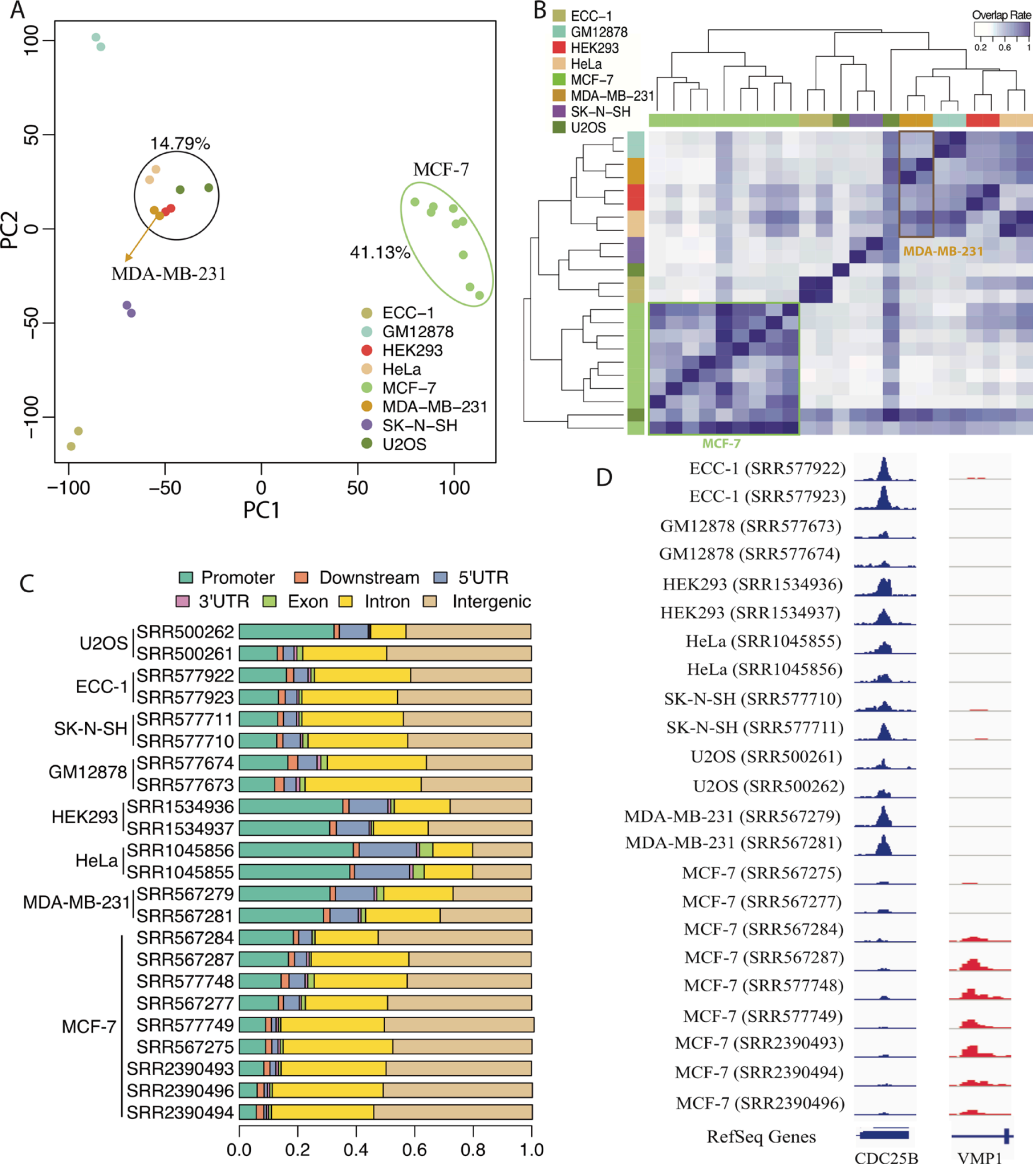
Comparison of FOXM1 binding events in different cell lines (**A**) PCA analysis of the normalized binding signal of FOXM1 in different ChIP-seq experiments. Colored dots represent different ChIP-seq experiments. The first PC explains 41.13% variation and the second PC explains 14.79% variation. (**B**) Peak overlap analysis based on the called binding peak in different ChIP-seq experiments. The color bars in left and top represent different ChIP-seq experiments. (**C**) Genomic regions distribution of FOXM1 binding peaks in different ChIP-seq experiments. (**D**) Two specific examples of FOXM1 binding.

We first sought to investigate the difference in FOXM1 binding events between different cell types. We divided the whole human genome into 100 bp bins, and for each bin we calculated the normalized binding signal (mean coverage per million reads) based on the continuous-valued TF binding signal provided in the bedGraph files. Bins with non-zero signal in at least one of the 23 ChIP-seq profiles were selected for PCA analysis. The results showed that all MCF-7 cell ChIP-seq profiles clustered together according to the first principle component, whereas MDA-MB-231 profiles were grouped with HEK293, HeLa and U2OS (Figure 2A). This is interesting since MDA-MB-231 and MCF-7 are both breast cancer cell lines, yet FOXM1 binding profiles in MDA-MB-231 are more similar to those in non-breast cell lines.

Second, we called FOXM1 binding peaks for all ChIP-seq experiments using Model-based Analysis of ChIP-Seq (MACS2) [56]. The number of FOXM1 binding peaks ranged from 517 to 54,916 depending on sequencing depth and other experimental factors, with details shown in Table 1. We examined the number of shared peaks between each pair of TF binding experiments. A peak in one experiment is counted as shared if there is at least a 1 bp overlap with peaks from the other experiment. Peak overlap analysis showed that most FOXM1 binding peaks called in MCF-7 cells are shared, despite the variation in peak numbers across different experiments (Figure 2B). As expected, peaks in MCF-7 exhibited low overlap with those called in the other cell types. Strikingly, binding peaks in MDA-MB-231 cells exhibited greater overlap with those in non-breast cell lines than with MCF-7 cells (Figure 2B). Moreover, FOXM1 binding peaks displayed different genomic distribution in different cell types (Figure 2C and see Supplementary Figure 1). Compared to other cell types, a smaller fraction of FOXM1 binding peaks in MCF-7 cells were observed in promoter regions, but more binding events occurred in intergenic regions. In MDA-MB-231 and other non-breast cell lines, FOXM1 binding signals exhibited high enrichment in regions proximal to transcription start sites (TSS) (from −1 kb to 1 kb) (see Supplementary Figure 2). Conversely, binding signals in MCF-7 cells were more enriched in regions distal to gene TSSs (from −3 kb to −2 kb and from 2 kb to 3kb).

Figure 2D shows a strong FOXM1 binding peak associated with the gene *CDC25B*, a well-known FOXM1 target gene [57], which was detected in all cell types but MCF-7. This phenomenon was also observed for many other cell cycle genes (data not shown), implying that FOXM1 may be less involved in cell cycle regulation in MCF-7 cells. In contrast, a FOXM1 binding peak associated with *VMP1*, a gene encoding a vacuole membrane protein [58], was only detected in MCF-7 cells (Figure 2D). Taken together, these results suggest that FOXM1 binding in MCF-7 cells differs from that of the other cell types, most notably MDA-MB-231. Moreover, FOXM1 binding in MDA-MB-231 was more similar to binding in non-breast cell lines. One plausible explanation is that estrogen receptor alpha (ERα), which is present in MCF7 but not in MDA-MB-231 cells, modulates FOXM1 activity by recruiting FOXM1 to ERα binding sites [59, 60] and causing differential FOXM1 binding between the two breast-cancer cell lines [59].

### Comparison of motifs enriched in FOXM1 binding sites

**Figure 3:**
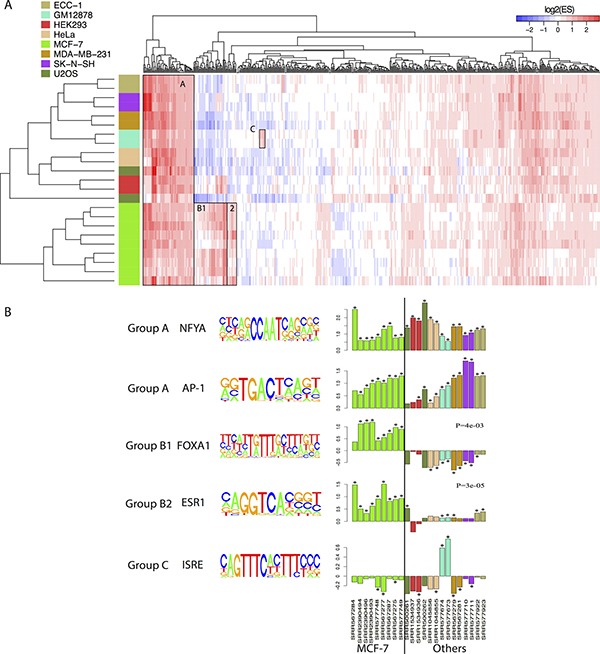
Comparison of enriched motifs of FOXM1 in different cells (**A**) Motif enrichment analyses across all FOXM1 ChIP-seq experiments. The color bars in the left represent different cell lines. The value in heatmap is log 2 transferred enrichment score (ES). (**B**) Five specific examples show the different binding of FOXM1 in different ChIP-seq experiments. Barplot was performed in log2 transferred enrichment scores. * represents the significance of enrichment (FDR < 0.01). Mann Whitney Wilcoxon Test *p*-value was showed in the FOXA1 and ESR1 examples.

To further investigate differences in FOXM1 binding events, we identified the TF binding motifs that are enriched in FOXM1 binding peaks in different cell lines. Motif enrichment analysis was conducted by scanning for 687 motifs from the TRANSFAC [53] and JASPAR [54] databases in peak regions (see details in Methods). To test whether a motif is enriched, we calculated enrichment scores for each FOXM1 binding peak across all the motifs enrolled in those two datasets. A log_2_-transformation was applied after performing the motif enrichment analysis so that a positive value indicates enrichment and a negative value indicates depletion. The results showed that experiments in MCF-7 cell lines (green bar) clustered together based on their enriched motifs compared with the other cell lines, whereas those from MDA-MB-231 (brown bar) cell lines clustered with other non-breast cells (Figure 3A).

In Figure 3A, we highlighted three interesting groups of enriched motifs. Group A contained the motifs enriched in all cell lines. Amongst these common motifs, NFYA (NFY CCAAT) has already been confirmed to be enriched in U2OS [47] and HeLa [61] cells; FOS and AP1 were previously shown to be associated with both ERα and FOXM1 binding [61, 62]; and BACH1 and BACH2 are two FOXM1 related proteins [63]. This group also contained some members of the E2F family of TFs involved in the cell cycle [64]. Group B consisted of two groups, where group B1 was mainly enriched in MCF-7 cells while group B2 was specific to MCF-7 cells. Specifically, motifs in group B1 consisted of diverse forkhead family and GATA family motifs, including FOXA1, FOXA2 and GATA3 which have been shown to act as ERα pioneer factors [65–67]. On the other hand, group B2 contained motifs associated with many kinds of receptors including estrogen receptor, nuclear receptor, peroxisome proliferator-activated receptor and thyroid hormone receptor. In group B2, ESR1 and ESRRA are two specific motifs for ERα [68, 69], and RORA (nuclear receptor related) and PPARG (peroxisome proliferator-activated receptor related) have been shown to associate with ERα [70, 71]. Group C contained motifs that are specifically enriched in GM12878 cells, and motifs which are associated with signal transduction, activation of transcription, and interferon regulatory factors.

Moreover, we compared log_2_-transformed ESs of five different motifs between the cluster groups including NFYA, AP-1, FOXA1, ESR1, and ISRE (Figure 3B). As shown, FOXA1 (Mann Whitney Wilcoxon Test *P* = 4e-03) and ESR1 (Mann Whitney Wilcoxon Test *P* = 3e-05) were significantly enriched in MCF-7 cell lines compared to others (Figure 3B). The same result was also observed for other ER-related motifs (see Supplementary Figure 3). These findings suggest that ERα may interact with FOXM1 and mediate FOXM1 binding in MCF-7 cell line.

Moreover, we conducted a preliminary exploration into other co-factors that may modulate FOXM1 binding activity. Due to higher enrichment in non-MCF-7 cells, we used NFH3 (see Supplementary Figure 4), a FOXM1 motif included in the TF Encyclopedia dataset [72] as the primary motif for SpaMo algorithm [73]. Besides, we utilized HOCOMOCO V10 [74], a human motif database as the secondary motif database as SpaMo input. Our results (see Supplementary Table 1) suggest that the motif of STAT3, a regulator involved in signal transduction and activation of transcription [75], was enriched in all cell lines except GM12878.

### Comparison of FOXM1 target genes

**Figure 4:**
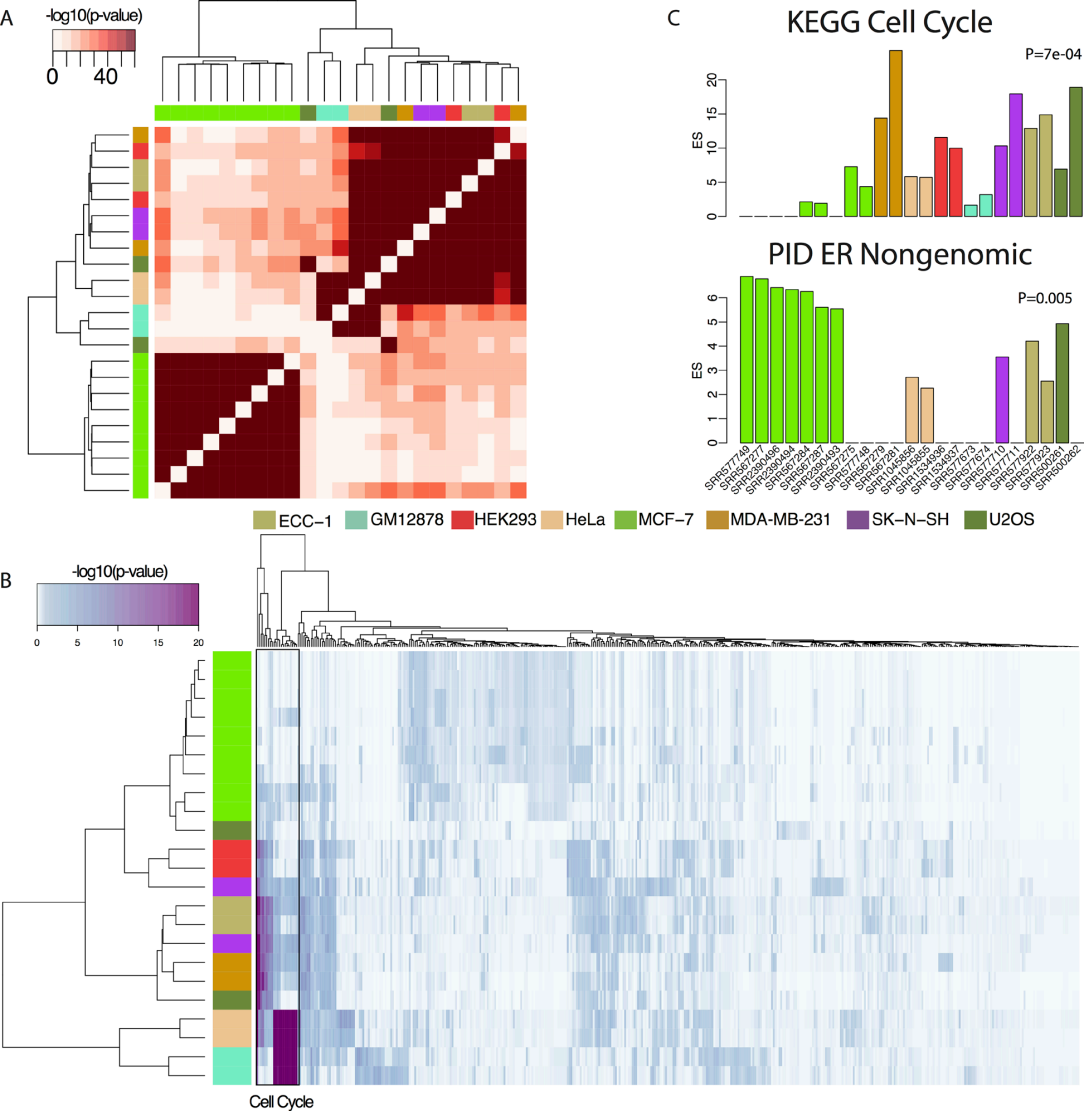
Comparison of target genes of FOXM1 in different cells (**A**) Heatmap of the enrichment of the target genes of pair-wised ChIP-seq experiments. The color bars around the heat map represent different cells. (**B**) Heatmap of pathway enrichment results based on negative log 10 transferred *p*-value. The color bars in the left represent different cells. (**C**) Enrichment scores comparison of two pathways, the cell cycle and the ER nongenomic, in MCF-7 and other cell lines. Colored bars represent corresponding cells. Mann Whitney Wilcoxon Test *p*-value was showed.

Next, we explored whether the differential genome-wide binding sites of FOXM1 results in the regulation of different target genes across varied cell lines. To identify the target genes of FOXM1, we applied a probabilistic model, TIP [76], to determine target genes for each ChIP-seq experiment (see Supplementary Table 2). The numbers of identified target genes for each ChIP-seq experiment were shown in Table 1, with a range of 92 (in MCF-7 cell) to 274 (in ECC-1 cell). According to the target genes, pair-wised enrichment analyses were conducted to calculate the corresponding *p* values. Based on negative log 10 transferred *p* values, the cluster results showed that target genes from all MCF-7 related ChIP-seq experiments exhibit significant overlap with each other but little overlap with those from other cells (Figure 4A). Consistent with our binding peaks and motif analyses, target genes detected in MDA-MB-231 and other non-breast cell lines displayed highly degree of consistency.

Furthermore, we performed comprehensive pathway analysis on target genes in each ChIP-seq experiment using the Molecular Signatures database (MSigDB) [77] C2 pathway database which integrates KEGG [78], REACTOME [79], Pathway Interaction Database (PID) [80] and BioCarta [81] databases. Because FOXM1 is a critical cell cycle regulating TF [82], we expected that the most significant enriched pathways would be related to the cell cycle. Indeed, our results showed that pathways related to the cell cycle were significantly enriched in FOXM1 target genes in MDA-MB-231 and other non-breast cell lines (Figure 4B). However, pathways enriched in FOXM1 target genes from MCF-7 cell lines exhibited lower enrichment in the cell cycle pathway (Figure 4C, Mann Whitney Wilcoxon Test *P* = 7e-04) but higher enrichment in an estrogen receptor related pathway, ER non-genomic pathway (Figure 4C, Mann Whitney Wilcoxon Test *P* = 0.005), when compared to other cell lines (Figure 4B). These results suggest that recruitment by ERα modifies the binding of FOXM1 in MCF-7 cell lines resulting in distinctive binding sites that differ from that of MDA-MB-231 and other non-breast cell lines. Moreover, the target genes of FOXM1 displayed a high degree of concordance between MDA-MD-231 and the other 6 non-breast cell lines even though they are derived from diverse tissues.

### Prognostic prediction based on FOXM1 activity in breast cancer

Several studies have shown that the transcriptional activity of FOXM1 is more predictive than its mRNA expression in cancers [43, 48–50]. To further investigate whether the different binding of FOXM1 is able to disturb its activity, we applied a computational method that infers FOXM1 activity based on its target gene expression, and examined whether it is prognosticative. We performed our analysis in the METABRIC breast cancer dataset, which contains gene expression and clinical profiles for 1,992 breast cancer patients [83]. We first merged target genes identified in each cell line into a core set of FOXM1 target genes and employed the BASE algorithm [55] to calculate an individual Regulatory Activity Score (iRAS) for each tumor sample based on the expression of FOXM1 target genes in the tumor gene expression profile. A higher iRAS in a tumor sample indicates that target genes of FOXM1 tend to have higher expression level and therefore a higher transcriptional activity of FOXM1 in this sample. Since FOXM1 target genes have been identified in 8 cell lines, we inferred FOXM1 activities in breast cancer samples using target genes identified in each individual cell line, resulting in eight iRASs for a tumor sample. Here, we utilized the target genes from the two breast cancer cell lines, MCF-7 and MDA-MB-231, as an example to investigate whether the inferred TF activities are associated with cancer outcome. Specifically, we denote FOXM1 activity inferred based on target genes identified in MCF-7 cell line as iRAS_MCF-7_. Similarly, iRAS_MDA_ indicates FOXM1 activity inferred based on its target genes in MDA-MB-231 cell line.

**Figure 5:**
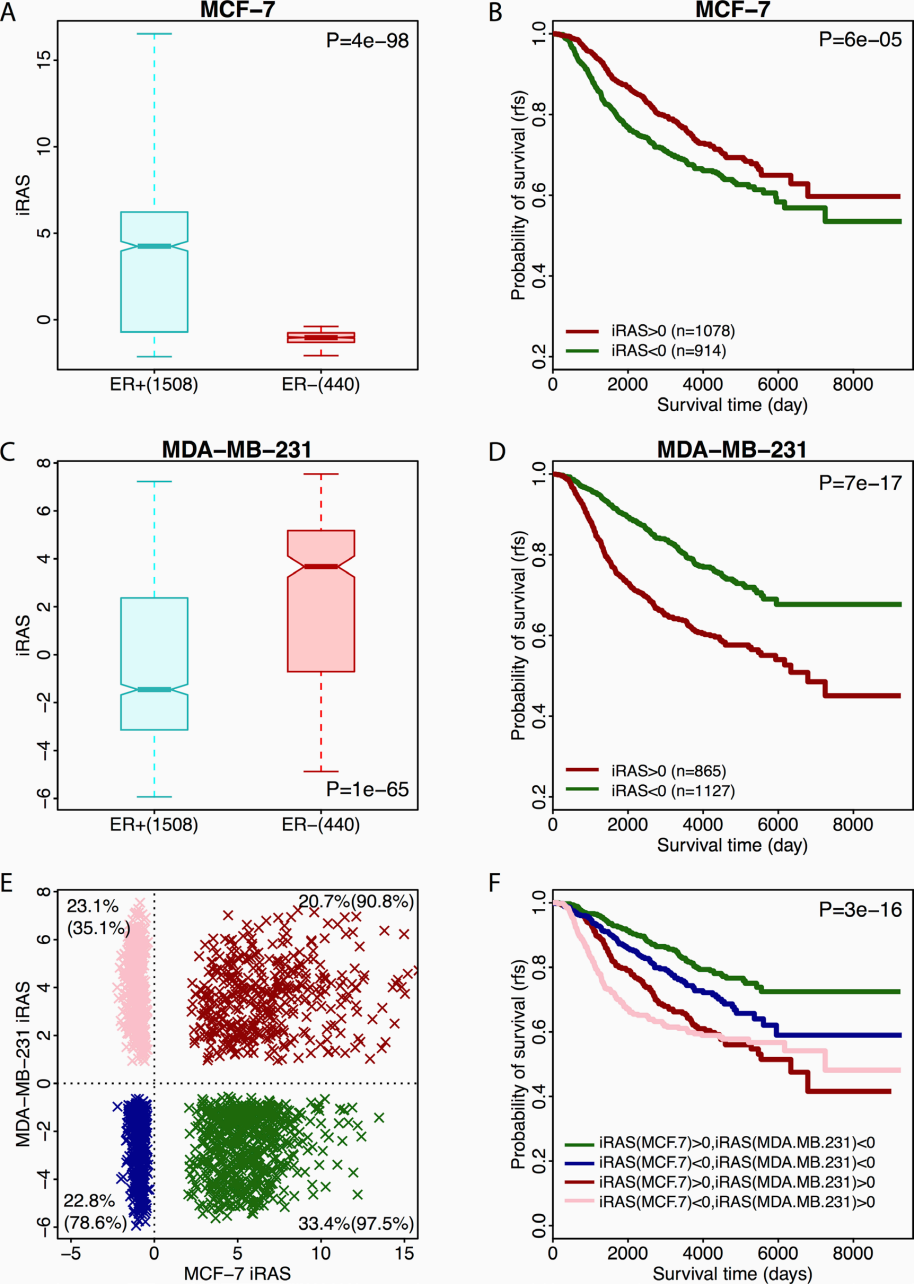
Associations between different FOXM1 activities and primary breast cancer sample prognosis (**A**) Boxplot for iRAS_MCF-7_ of ER+ and ER- patients. (**B**) Survival curve for ER+ and ER- patients with high or low iRAS_MCF_. (**C**) Boxplot for iRAS_MDA_ of ER+ and ER- patients. (**D**) Survival curve for ER+ and ER- patients with high or low iRAS_MDA_. (**E**) Distribution of patients based on both iRAS_MCF-7_ and iRAS_MDA_. Red dots: patients with both positive iRAS_MCF-7_ and iRAS_MDA_. Blue dots: patients with both negative iRAS_MCF-7_ and iRAS_MDA_. Green dots: patients with positive iRAS_MCF-7_ and negative iRAS_MDA_. Pink dots: patients with negative iRAS_MCF-7_ and positive iRAS_MDA_. The percentages at four corners are the fractions of patients with ER+ in the corresponding group. The number 1~4 mapped to the survival curves. (**F**) Survival curves for the four patient groups.

We first compared the inferred regulatory activities of FOXM1 in ER-positive (ER+) versus ER-negative (ER−) breast tumor samples. When FOXM1 targets identified in MCF-7 were used for activity inference, the resulting iRAS_MCF-7_ showed significant difference between ER+ and ER− samples (Figure 5A). As shown, ER+ samples exhibited significantly higher iRAS_MCF-7_ than ER- samples (Mann Whitney Wilcoxon Test *P* = 4e-98). However, when we stratified patients into ER+ and ER- groups, iRAS_MCF-7_ is not associated with prognosis any more (*P* > 0.1). This indicates that FOXM1 targets in MCF-7 cell lines reflect estrogen receptor activity in breast tumor samples, implying that ERα serves as the major factor that mediates FOXM1 genomic binding in ER+ breast cancer. Namely, in majority binding sites, ERα mediates FOXM1 through its DNA motifs, and thus regulating the transcription of ERα target genes. We further divided patients into two groups based on iRAS_MCF-7_, patients with high iRAS_MCF-7_ (iRAS_MCF-7_ > 0) and those with low iRAS_MCF-7_ (iRAS_MCF-7_ < 0), and compared their prognosis. Patients with high iRAS_MCF-7_ exhibited better prognosis compared to those with low iRAS_MCF-7_ (Figure 5B, log-rank *P* = 6e-05). These observations further implied that iRAS_MCF-7_ primarily reflects ERα activity in a tumor sample where higher ERα activity indicates greater sensitivity to hormone treatment, even though the major function of FOXM1 is to regulate cell cycle division.

We then examined the regulatory activity of FOXM1 inferred based on its targets in MDA-MB-231, an ER-negative breast cancer cell line, and generated the corresponding iRAS_MDA_. We observed significantly lower iRAS_MDA_ in ER+ than in ER- tumor samples (Figure 5C, Mann Whitney Wilcoxon Test *P* = 1e-65), which is opposite to what we observed for iRAS_MCF-7_. Furthermore, patients with low iRAS_MDA_ had better prognosis compared to those with high iRAS_MDA_ (Figure 5D, log-rank *P* = 7e-17). These results suggested that iRAS_MDA_ reflects the activity of FOXM1 in regulating cell proliferation. Lower iRAS_MDA_ indicates slower proliferation of tumor cells in a patient's sample, and therefore informs better prognosis. The relevance of iRAS_MDA_ with cell proliferation was further confirmed by its high positive correlation with the E2F4 score (Pearson Coefficient *R* = 0.93, see Supplementary Figure 5), which is calculated based on an E2F4 gene signature that serves as an accurate indicator of cell proliferation in breast tumor cells [84]. In contrast, iRAS_MCF-7_ inferred based on FOXM1 targets in MCF-7 cell lines showed no significant correlation with iRAS_E2F4_ (Pearson Coefficient *R* = 0.017, see Supplementary Figure 5). However, the calculated iRAS_MCF-7_ had high positive correlation (Pearson Coefficient *R* = 0.45, see Supplementary Figure 5) with *ESR1* gene expression while iRAS_MDA_ was negatively correlated (Pearson Coefficient *R* = −0.34, Supplementary Figure 5) with *ESR1* gene expression. Moreover, we also inferred the FOXM1 activities in breast tumor samples based on its target genes identified in the other six non-breast cell lines. As expected the resulting iRAS were similar to iRAS_MDA_ but different from iRAS_MCF-7_ in terms of prognostic prediction (Supplementary Figures 5 and 6).

Our observations indicate that FOXM1 activity inferred based on its targets in MCF-7 cell line serves as a proxy for ERα activity, while iRAS_MDA_ and other iRAS calculated using the target genes in non-breast cells serve as proxies for proliferation. Since both ER status and proliferation are critical factors for determining patient prognosis in breast cancer, we postulated that prognosis could be better predicated based on a combination of iRAS_MCF-7_ and iRAS_MDA._ Specifically, we divided the patients into four categories based on both iRAS_MCF-7_ and iRAS_MDA_ (Figure 5E). For example, patients (green) with high iRAS_MCF-7_ and low iRAS_MDA_ were classified as group 1 which encompassed 33.4% of patients in the METABRIC dataset, 97.5% of which were ER+ patients. In contrast, patients (pink) in group 4 contained 23.1% of METABRIC patients with only 35.1% of these patients being ER+. This observation further suggests that the calculated iRAS_MCF-7_ reflects the activity of estrogen receptor, which is consistent with Figure 5A where ER+ patients exhibited higher iRAS_MCF-7_ than ER- patients. More interestingly, when we compared group 1 with group 4, we found that ER+ patients are more likely to maintain lower cell proliferation. We further found that patients in group 1 have the best outcome compared to the other 3 groups (Figure 5F, log-rank *P* = 3e-16), as these patients are associated with higher ERα activity and lower proliferation. Moreover, patients in group 1 and 2 had better prognosis compared to those in group 3 and 4 which implies that proliferation is more prognosticative than ER activity in breast cancer. These observations suggest that the combination of the inferred activities of both ERα and proliferation can provide more precise prognostic predictions in breast cancer.

## DISCUSSION

TFs play crucial roles in regulating gene expression by binding to many cis-regulatory elements that decide cell fate. ChIP-seq technology has been widely utilized to investigate the binding sites of several TFs in a panoply of biological contexts. Intuitively, we would expect to observe more similar genomic binding profiles of a TF in cell lines from the same or more closely related tissues. However, our analyses on FOXM1, a well-known cell cycle regulator [85], provide an example showing that the overall genetic similarity of cell lines does not always hold precedent over context-specific TF co-factors in determining TF binding profiles.

In this study, we focus on the binding events of FOXM1 using 23 ChIP-seq experiments from 8 human cell lines that encompass 7 different tissues (Table 1). We comprehensively examine the binding patterns of FOXM1 across 4 information layers including raw binding signals, binding peaks, target genes, and regulator activity (Figure 1). The PCA analysis of raw binding signals showed that ChIP-seq experiments in MDA-MB-231 clustered with HEK293, HeLa and U2OS cell lines signals while those from MCF-7 cell lines group together independently. This is surprising due to the fact that MCF-7 and MDA-MB-231 are both breast cancer cell lines that are dissimilar in their FOXM1 binding profiles. In addition, binding profiles in MDA-MB-231 are more similar to those from other non-breast tissues.

Peaks called in MCF-7 cell lines exhibited high overlap with each other even though the total number of peaks called differed between experiments. Contrastingly, peaks identified in another breast cancer cell line, MDA-MB-231, showed a high degree of overlap with other non-breast cell lines. Furthermore, utilizing the called binding peaks of FOXM1, we compared motifs enriched in different ChIP-seq experiments and demonstrated that the enriched motifs are highly overlapped between MCF-7 ChIP-seq experiments while those in MDA-MB-231 were extremely similar with that in the other non-breast cell lines. Notably, peaks called in MCF-7 cell lines were significantly enriched in ERα related motifs. Similar results are observed in the comparison of target genes as well. Specifically, genes identified in MCF-7 cell lines were highly redundant, whereas genes identified in MDA-MB-231 cell lines showed greater overlap with those found in non-breast cell lines. Additionally, the cell cycle pathway was highly enriched in FOXM1 target genes identified in MDA-MB-231 and non-breast cell lines, but not in MCF-7. However, the ER non-genomic pathway was significantly enriched in target genes identified in MCF-7 cell lines. Interestingly, ECC-1, an endometrial cell line that is also ER positive cells, shows similar pattern with other non-MCF-7 cell lines in terms of FOXM1 binding, suggesting that the effect of co-factor ERα on FOXM1 binding might be breast tissue-specific.

Based on these results, we suspected that FOXM1 and ERα might exhibit a physical functional interaction in MCF-7 cell lines which is consistent with reports from previous studies [59, 86, 87]. We hypothesized that this interaction alters the binding properties of FOXM1 in MCF-7 cell line and modulates FOXM1-driven transcriptional output. Sanders *et al.* [59] indicated that the recruitment effects of ERα on FOXM1 binding in ER positive breast cancer cell lines differs in ER negative cell lines. In our study, we demonstrate that the influence of ERα not only causes differences between the two breast cancer cell lines, but also induces differences between ER positive breast cancer cell lines and non-breast cancer cell lines. Moreover, the binding sites of FOXM1 in MDA-MB-231, an ER negative breast cancer cell line, and those in non-breast cell lines showed a high degree of overlap even though they are classified as different tissues. These results suggest that the binding of FOXM1 can be modulated by a co-factor such as ERα, and this interaction lead to significant changes in activated pathways that hold precedence over tissue-specificity.

Lastly, we further tested whether the varied binding affects the overall regulatory activity of FOXM1 in a cell type-specific fashion. In support of our earlier results, we found that the activity of FOXM1 is mainly captured by ERα binding events in MCF-7 cell line which reflects ERα activity. In contrast, the FOXM1 activity inferred using target genes in MDA-MB-231 or non-breast cell lines, is more indicative of cell proliferation. Both inferred activities are significantly predictive of breast cancer prognosis. Nevertheless, the results of multivariate Cox regression model suggested that the activity of FOXM1 in MDA-MB-231 cell line, proliferation activity, still is an independent biomarker in breast cancer sample prognosis (see Supplementary Table 3). While FOXM1 activity in MCF-7 cells is not associated with prognosis after considering clinical variables which is consistent with the result of ER status. Moreover, combining information about the activity of both estrogen receptor and cell proliferation activities results in a gene signature that predicts patient prognosis with high accuracy.

In light of our findings, we were surprised by how the influence of a single co-factor on the binding patterns of a certain TF could induce a completely different binding profile. The co-factor discovery analysis (see Supplementary Table 1) suggests that STAT3 might be a potential co-factor of FOXM1, in line with previous report that the activation of FOXM1 is dependent on STAT3 activity [88]. Because of the diversity and uncertainty of FOXM1 motifs (see Supplementary Figure 4), we considered this analysis as a preliminary exploration to provide further directions.

**Figure 6:**
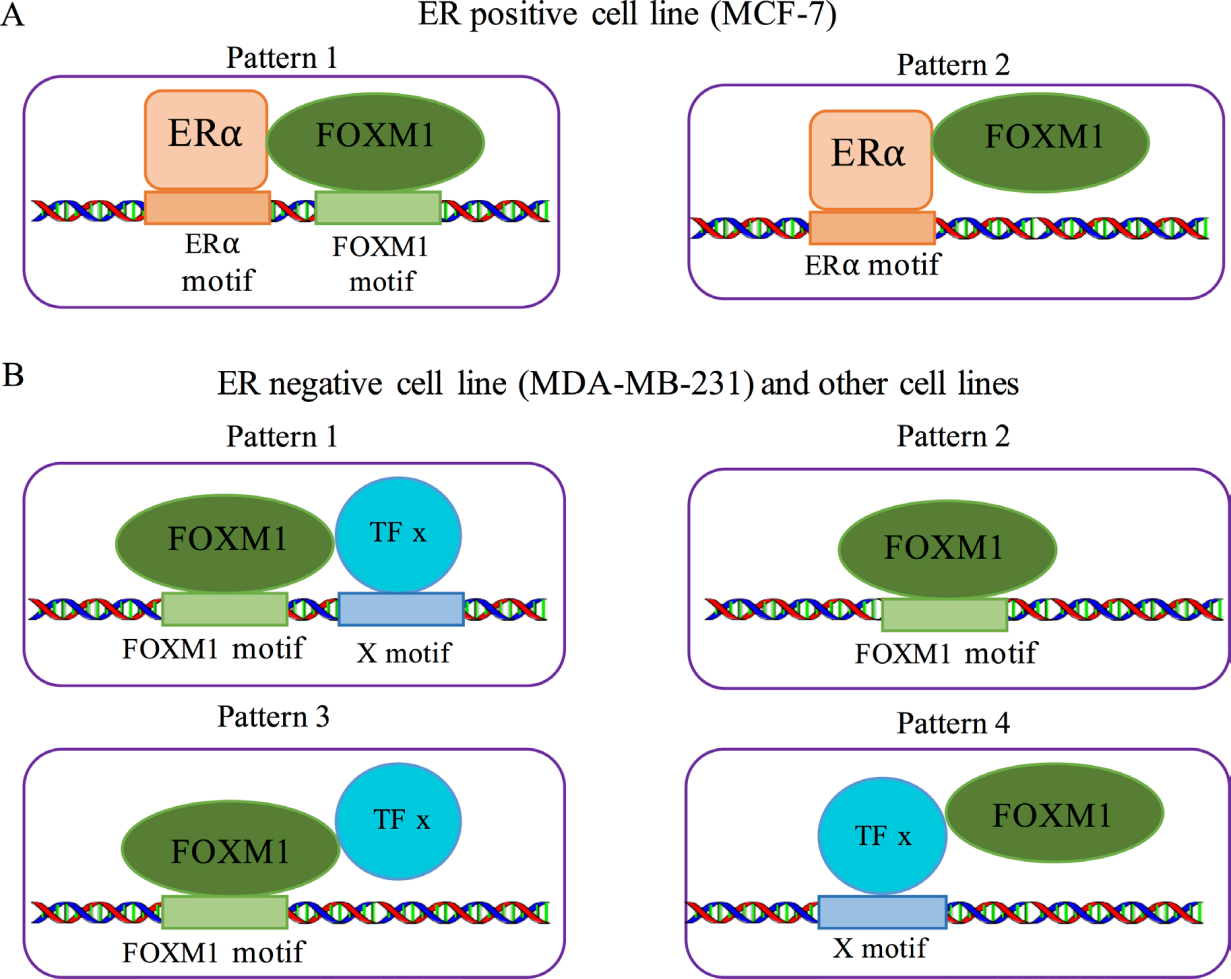
Co-binding patterns for FOXM1 in different cells (**A**) Two possible co-binding pattern of ERa and FOXM1 in MCF-7 cell lines. (**B**) Four possible co-binding patterns in MDA-MB-231 and other non-breast cell lines.

In view of our analyses, we inferred the binding patterns of FOXM1 in different cell lines based on its physical interactions with different co-factors. In MCF-7 cell lines FOXM1 binding might be mediated by ERα, supporting the following two possible patterns: (1) FOXM1 interacts with ERα and both bind their own motifs, eliciting a cooperative binding pattern, and (2) FOXM1 interacts with ERα but only ERα binds its DNA motif (Figure 6A). Our motif enrichment results (Figure 3A) indicate that FOXM1 binding sites in MCF-7 cells are enriched not only in ER-related motifs but also in cell cycle related motifs. This observation suggests that the cooperative binding (Figure 6A, pattern 1) is the more likely to be the binding pattern of FOXM1 in MCF-7, which is in agreement with the previous study by Sanders *et al.* [59]. In contrast, in MDA-MB-231 and the non-breast cell lines FOXM1 may have four possible binding patterns (Figure 6B). (1) FOXM1 interacts with another TF *x*, each binding with its own motif; (2) FOXM1 binds with its motif without interacting with a co-factor. (3) FOXM1 interacts with another TF *x,* but DNA binding is mediated by FOXM1 motif. (4) FOXM1 interacts with another TF *x* that binds with its motif. These four binding patterns may co-exist in MCF-7 and non-breast cancer cell lines. When ChIP-seq data for FOXM1 are generated in more cell lines, we may expect to observe in some cell lines that FOXM1 binding events are mainly mediated by the interaction of another TF *x* with its DNA motif as observed in MCF-7 cells.

Although the FOXM1 ChIP-seq data were collected from 6 different studies with different FOXM1 antibodies and sequence depths, we achieved consistent results according to the comparisons based on raw signals, binding peaks, motifs and target genes. Our systematic analyses revealed that binding of FOXM1 in MCF-7 cell line differs with MDA-MB-231 and other non-breast cell lines. Moreover, the binding of FOXM1 exhibited highly similarities in cell lines from MDA-MB-231 and six other different tissues. Our results provide new insights into TF binding and suggest that some TFs might not be as affected by tissue type as previously thought. However, there exists some limitations to our methods. First, noise caused by the hybridization process in ChIP-seq experiments[3] may influence the accuracy of our results. Second, the computational nature of our analysis is unable to directly reveal a direct PPI between ERα and FOXM1. However, a previous study by Sander *et al.* [59] revealed that ERα co-immunoprecipitates with FOXM1 demonstrating that they are able to interact *in vitro*. In summary, our analysis robustly demonstrated altered FOXM1 binding as a result of ERα activity and provided new insights concerning the binding modes of FOXM1 in different cell types.

## MATERIALS AND METHODS

### Collection of FOXM1 ChIP-seq experiments

We retrieved 23 FOXM1 ChIP-seq experiments from the NCBI GEO database [51] (Table 1). We downloaded two FOXM1 ChIP-seq experiments performed in ECC-1 cells (endometrium cancer cell line), two ChIP-seq experiments performed in GM12878 cells (blood cancer) and two ChIP-seq experiments performed in SK-N-SH cells (neuroblastoma cancer) from ENCODE using Series accession number GSE32465 [46]. Two FOXM1 ChIP-seq experiments in HEK293 cells (kidney cancer cell line) were acquired using Series accession number GSE60032 [44]. Two FOXM1 ChIP-seq experiments performed in HeLa cells were downloaded under Series accession number GSE52098 [45]. Two FOXM1 ChIP-seq experiments performed in U2OS cells (osteosarcoma cancer cell line) were downloaded under Series accession number GSE38170 [47]. Two FOXM1 ChIP-seq experiments performed in MDA-MB-231 cells (breast cancer cell line) were downloaded with Series accession number GSE40762 [59]. Nine FOXM1 ChIP-seq experiments performed in MCF-7 cells (breast cancer cell line) were downloaded under Series accession numbers GSE32465 [46], GSE72977 [43], and GSE40762 [59]. The antibodies used in GSE60032 and GSE72977 are GTX-102170 and SC-501, respectively. In addition, FOXM1 antibody SC-502 is used in the other experiments.

### Breast cancer database

In this study, we downloaded the METABRIC breast cancer dataset [83] to calculate iRAS and perform survival analysis with the accession number EGAS00000000083. This dataset contains gene expression profiles and clinicopathological data for 1,992 breast cancer samples including time-to-event information and ER status.

### ChIP-seq reads alignment

After collecting the FOXM1 ChIP-seq experiments, we first used NCBI Sequence Read Archive [89] to align the reads in each experiment. Fastq-dump was applied to convert the raw ChIP-seq experiment profiles to fastq files. We used Bowtie [90] to map reads to the UCSC hg19 human sequence and generated SAM files for each ChIP-seq experiment. We then sorted these SAM files using SAM tools [91] and then generated bedGraph files using BEDtools [92]. The resulting reads for each ChIP-seq experiment were listed in Table 1. The bedGraph files were further used to perform PCA analysis [52] and display specific peaks with Integrative Genomics Viewer [93].

### ChIP-seq peak calling and Genomic distribution of binding peaks

Using the generated SAM files, significant peaks were called by MACS2 [56] setting *q*-value cutoff as 0.01. The number of called peaks for each experiment were shown in Table 1. The peaks overlap coefficients were used to cluster ChIP-seq experiments. The Cis-regulatory Element Annotation System [94] was applied to functionally annotate binding peaks across the genome.

### Motif enrichment analysis

In our study, we utilized two high-quality transcription factor binding profile databases, TRANSFAC [53] and JASPAR [54], to perform the enrichment analysis. We used FIMO [95] to scan peak regions for occurrences of all the 687 motifs contained in the two databases. Second, we refined the called peaks using the summit files generated by MACS [56]. Specifically, for a given peak, we set up a region surrounding its summit (from −250 to 250 of summit). Then, the overlapped district of the peak and this region was considered as the refined peak. To perform the enrichment analysis, we set up two control regions (the same length of the refined peak) for each refined peak. One is upstream of the refined peak and ends at the start position of the refined peak. The other is downstream of the refined peak and starts at the ending position of the refined peak. Third, enrichment score (ES) was calculated and corresponding *p* value was calculated using Chi-squared test. The function heatmap.2 from the R package “gplots” was used to show the motif enrichment analysis results after log 2 transformation of the ES.

### Identification of target genes

We used the TIP algorithm [76] to identify the target genes of FOXM1 for different ChIP-seq experiments (bedGraph files) by assigning each gene a probability of being bound by FOXM1 based on the averaged background binding signal. An FDR threshold of 0.01 was used to identify putative target genes of FOXM1 for each ChIP-seq experiment. The number of target genes identified in each experiment are shown in Table 1 and the specific genes are listed in an additional table (see Supplementary Table 1). Moreover, the average signal of FOXM1 binding in the DNA region surrounding TSS profiles were generated as the output of the TIP algorithm. These profiles were applied to show the FOXM1 binding distribution around gene TSSs (from −3 kb to 3 kb of TSS).

### Pathway enrichment analysis

In our study, we used the MSigDB [77] C2 pathway database to perform pathway enrichment analysis. The C2 database integrates 4 independent pathway datasets including REACTOME, PID, BIOCARTA, and KEGG [78–81]. We excluded pathways containing less than 40 target genes and calculated the ES and *p*-value for all FOXM1 target gene sets using a two-sided hypergeometric test. Clustering analysis of pathway enrichment was performed by log10-transforming the enrichment *p*-values and assigning a positive value if the corresponding ES was < 1 and a negative value if the ES was > 1. All *p*-value calculations were performed using the R “Hypergeometric” package and clustering analysis was implemented using the “gplots” package in R.

### Calculation of FOXM1 activity

According to the target gene sets identified using our previously TIP algorithm [76], we first merged target genes identified in each cell line into a core set of FOXM1 binding affinity data. Specifically, g_j_ = {g_1,j_, g_2,j_, …g_i,j_, …, g_n,j_}, where g_j_ is the target gene set for cell line *j*, g_i,j_ is the ith gene in whole human genome, n is the total number of whole human genome genes. g_i,j_ is 1 suggests that the ith gene is FOXM1 target gene in cell line *j*, whereas, the ith gene is non-target gene. Integrating breast cancer samples gene expression profiles, we employed BASE [55], a rank-based algorithm, to infer an iRAS for each breast tumor. Briefly, for a given sample S = {s_1_, s_2_, …, s_i_, …, s_n_}, where s_i_ is expression level of the ith gene. Basing on the FOXM1 binding affinity data, BASE calculated a cumulative distribution function for both target genes and non-target genes, denoting as T(i) and Non-T(i). The maximum deviation between these two functions infers the similarity score between the binding affinity data and sample gene expression. After 1000 times random permutation, we normalized those scores and got the iRAS which infers the activity of FOXM1 in a specific sample.

### Survival analysis

The Kaplan-Meier method was applied to compare survival prognosis of patients in different groups and the log-rank test was used to calculate the *p*-value. Multivariate cox regression model was used to examine the independent prognostic power of iRAS considering important clinical variables including age, tumor stage, tumor grade, ER status, PR status and HER2 status. The R package “survival” was used to implement survival analysis.

## SUPPLEMENTARY MATERIALS FIGURES AND TABLES







## Abbreviations

TF: transcription factor
ERa: estrogen receptor alpha
ER: estrogen receptor
ER+: ER-positive
ER-: ER-negative
ChIP-chip: chromatin immunoprecipitation followed by microarray hybridization
ChIP-seq: chromatin immunoprecipitation followed by high-throughput DNA sequencing
ENCODE: Encyclopedia of DNA Elements
GEO: Gene Expression Omnibus
PCA: principle component analysis
BASE: Binding Association with Sorted Expression
MACS: Model-based Analysis of ChIP-Seq
TSS: transcription start sites
MSigDB: Molecular Signatures database
iRAS: individual Regulatory Activity Score
ES: enrichment score
