# Supplementary material for: Cancer cell line specific co-factors modulate the FOXM1 cistrome
Source: Oncotarget. 2017 Aug 24;8(44):76498–515. doi: 10.18632/oncotarget.20405 (PMC5652723; doi:10.18632/oncotarget.20405)
Supplement: Supplementary file 1 [file oncotarget-08-76498-s001.pdf]

Cancer cell line specific co-factors modulate the FOXM1 cistrome

SUPPLEMENTARY MATERIALS

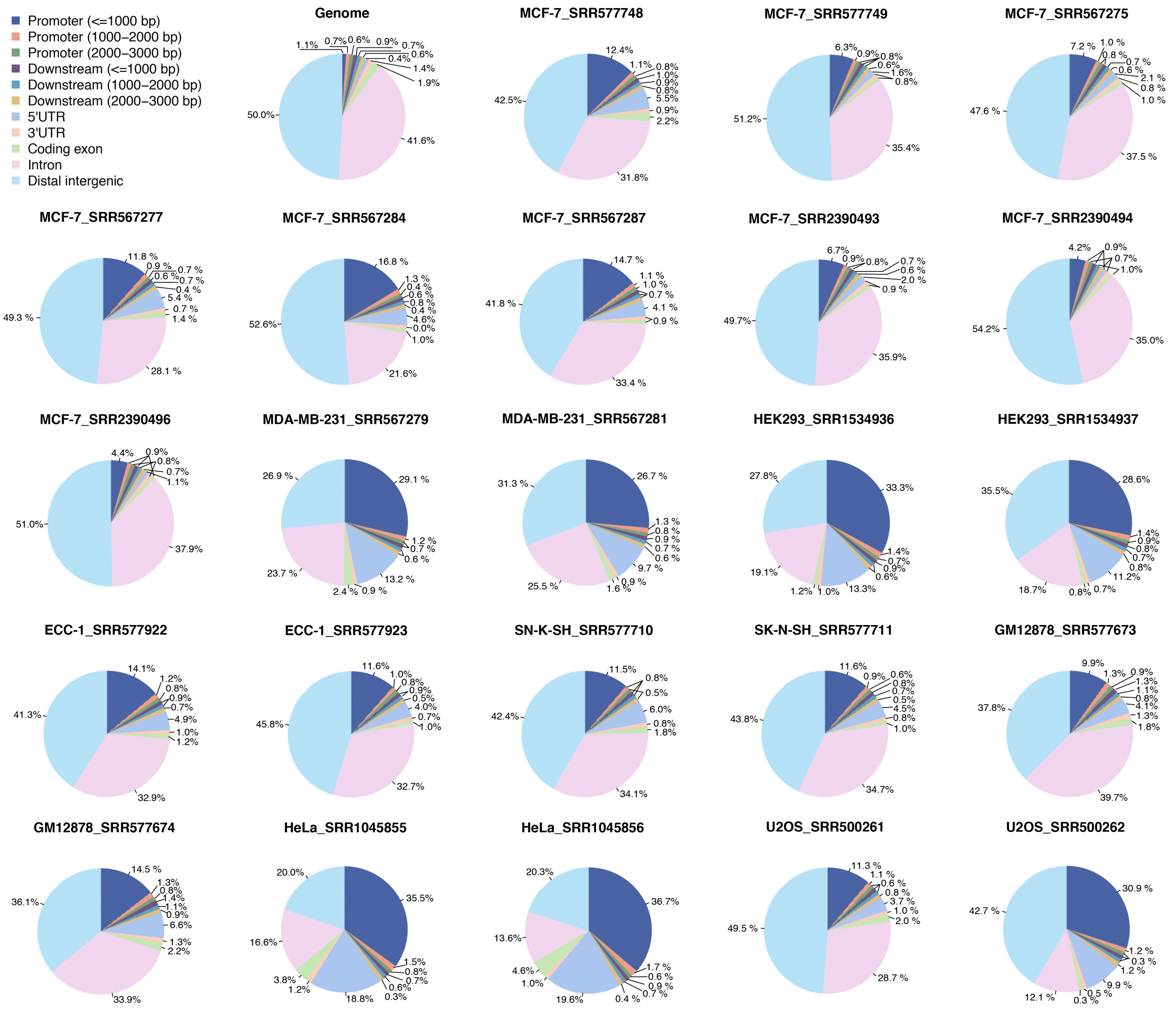

Supplementary Figure 1: The detail genomic distribution of FOXM1 binding peaks in different ChIP-seq experiments. Each color maps a region (Top left). The region fractions for whole genome was provided as reference.

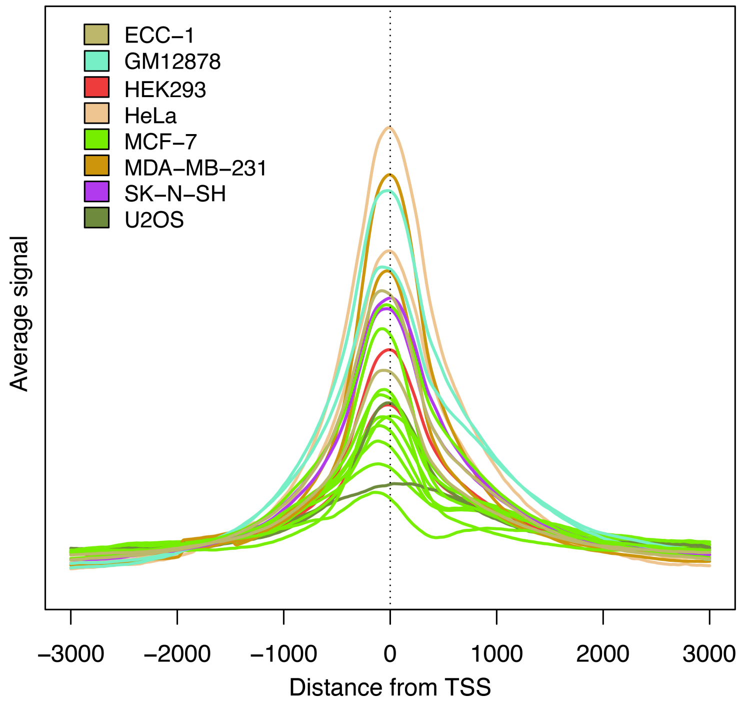

Supplementary Figure 2: The distribution of FOXM1 average binding signal in the DNA region surrounding transcription start site. Different colors represent different cell lines. We tested the binding signals around transcription start sites (TSS) from downstream 3 kb to upstream 3 kb.

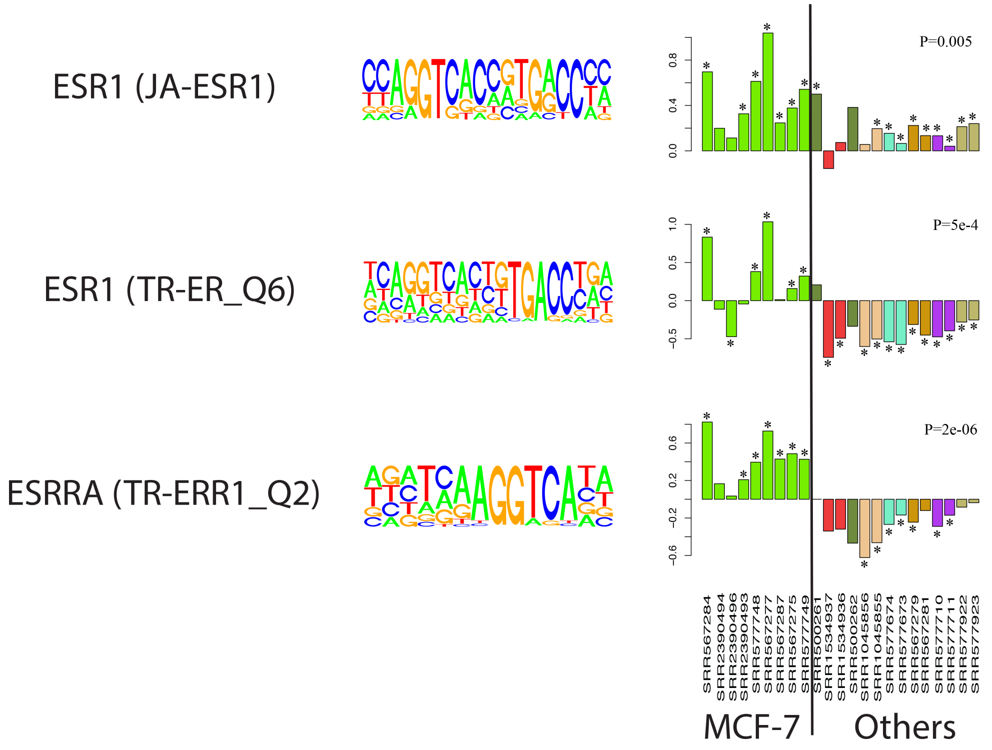

Supplementary Figure 3: Enrichment results of different FOXM1 ChIP-seq experiments in ERa related motifs. The first column list the name of ERa and its corresponding name in the JASPAR (JA) or TRANSFAC (TR) databases. The second column is the logo of corresponding motifs. The third column shows the enrichment results. Barplot was performed in log2 transferred enrichment scores. * represents the significance (FDR < 0.01) of enrichment. Mann Whitney Wilcoxon Test p-value was showed in the barplot to show the difference between MCF-7 and other cell lines.

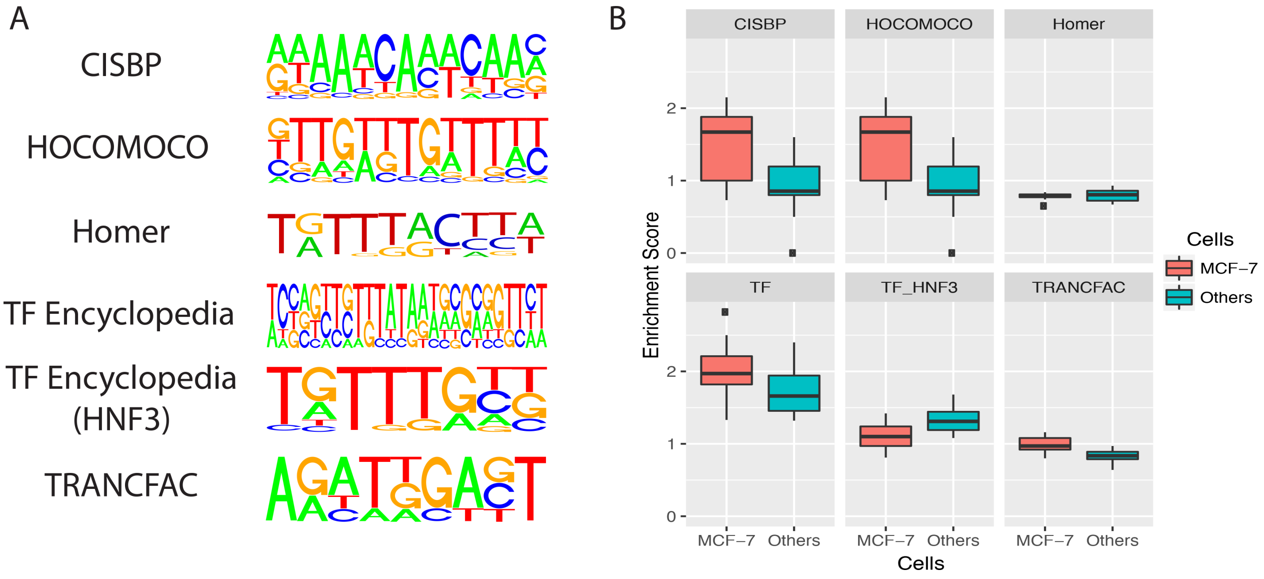

Supplementary Figure 4: Comparison of known FOXM1 motifs. We collected 6 known FOXM1 motifs from 5 different motif databases, CISBP [5], HOCOMOCO [3], Homer [2], TF Encyclopedia [8] and TRANSFAC [6] as shown in panel (**A**), to identify the strongest FOXM1 motif. We repeated the motif enrichment analysis with these 6 FOXM1 motifs across those 23 FOXM1 ChIP-seq experiments. The result showed that the NHF3, enrolled in the TF Encyclopedia database, exhibited higher enrichment scores in MDA-MB-231 and other nonbreast cell lines than MCF-7 out of all the FOXM1 motifs (**B**). Moreover, this motif showed the highest similarity to the RYAAAYA (Q-value = 3e-04) conserved FOXM1 consensus [4, 7] according to the TomTom motif comparison tool [1], suggesting that it is most likely to be the actual FOXM1 motif. Therefore, we used the NFH3 as the primary motif of the co-factor analysis.

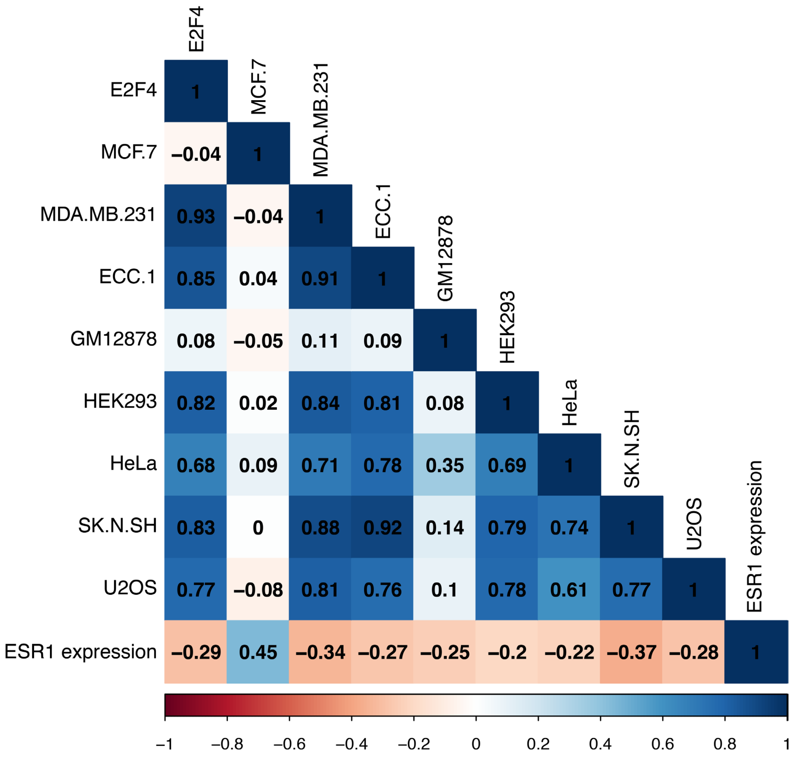

Supplementary Figure 5: Correlation between ESR1 expression, E2F4 activity and different FOXM1 activities in breast cancer samples. The color scale from red (negative correlation) to blue (positive correlation). The corresponding correlation value is shown in each cell. The higher correlation the bluer. The lower correlation the more red.

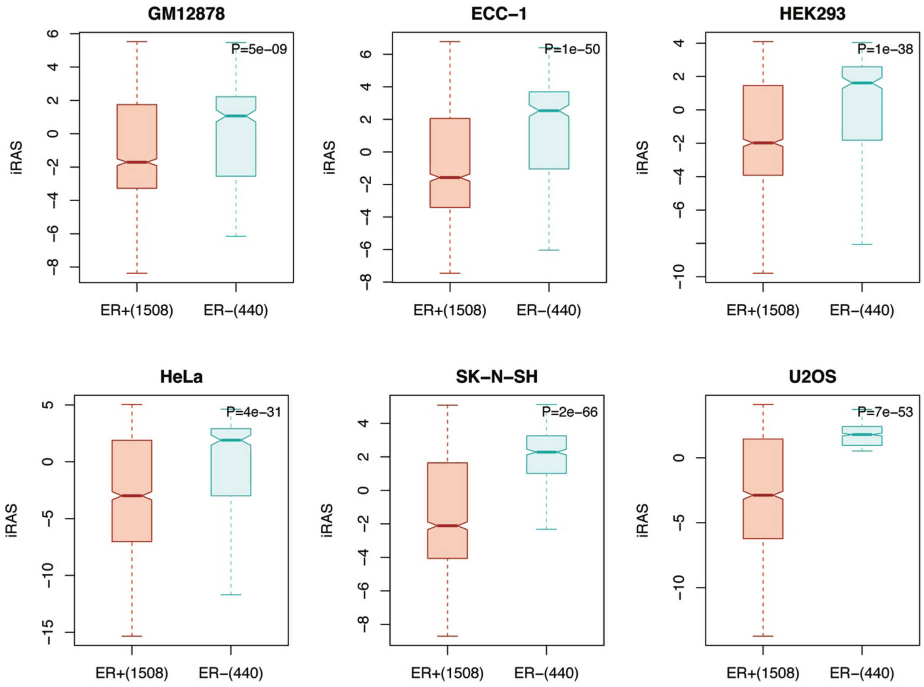

Supplementary Figure 6: Boxplot for FOXM1 activity scores for different cells. Each box showed the distribution of iRAS (FOXM1 activity score) of ER+ and ER− patients in which the iRAS was calculated using the target genes in corresponding cell (above the box). Mann-Whitney *U*-test *P*-values are showed.

Supplementary Table 1: The target genes of FOXM1 identified in each ChIP-seq experiment. See Supplementary_Table_1

Supplementary Table 2: The co-factors of FOXM1 identified in each cell line. See Supplementary_ Table_2

Supplementary Table 3: The Cox regression results for iRAS in MCF-7 and MDA-MB-231 cells considering clinical factors

Cell line	Class	*P* value	Coef	95% CI
MCF-7	iRAS	2.E-01	0.98	0.95–1.01
	Age	5.E-01	1.00	0.99–1.01
	Stage	1.E-01	1.09	0.98–1.21
	Grade	1.E-04	1.44	1.19–1.74
	ER + vs. −	6.E-01	0.93	0.69–1.26
	PR + vs. −	3.E-02	0.76	0.60–0.98
	Her2 + vs. −	5.E-05	1.73	1.33–2.27
MDA-MB-231	iRAS	3.E-07	1.09	1.06–1.13
	Age	4.E-01	1.00	0.99–1.01
	Stage	1.E-01	1.09	0.98–1.21
	Grade	1.E-01	1.20	0.98–1.47
	ER + vs. −	8.E-01	0.97	0.74–1.28
	PR + vs. −	1.E-01	0.82	0.64–1.05
	Her2 + vs. −	2.E-04	1.63	1.26–2.12

REFERENCES

1. Gupta S, Stamatoyannopoulos JA, Bailey TL, Noble WS. Quantifying similarity between motifs. Genome Biol. 2007; 8:R24.
2. Heinz S, Benner C, Spann N, Bertolino E, Lin YC, Laslo P, Cheng JX, Murre C, Singh H, Glass CK. Simple combinations of lineage-determining transcription factors prime cis-regulatory elements required for macrophage and B cell identities. Mol Cell. 2010; 38:576–589.
3. Kulakovskiy IV, Medvedeva YA, Schaefer U, Kasianov AS, Vorontsov IE, Bajic VB, Makeev VJ. HOCOMOCO: a comprehensive collection of human transcription factor binding sites models. Nucleic Acids Res. 2013; 41:D195–202.
4. Major ML, Lepe R, Costa RH. Forkhead box M1B transcriptional activity requires binding of Cdk-cyclin complexes for phosphorylation-dependent recruitment of p300/CBP coactivators. Mol Cell Biol. 2004; 24:2649–2661.
5. Weirauch MT, Yang A, Albu M, Cote AG, Montenegro-Montero A, Drewe P, Najafabadi HS, Lambert SA, Mann I, Cook K et al. Determination and inference of eukaryotic transcription factor sequence specificity. Cell. 2014; 158:1431–1443.
6. Wingender E, Dietze P, Karas H, Knuppel R. TRANSFAC: a database on transcription factors and their DNA binding sites. Nucleic Acids Res. 1996; 24:238–241.
7. Ye H, Kelly TF, Samadani U, Lim L, Rubio S, Overdier DG, Roebuck KA, Costa RH. Hepatocyte nuclear factor 3/fork head homolog 11 is expressed in proliferating epithelial and mesenchymal cells of embryonic and adult tissues. Mol Cell Biol. 1997; 17:1626–1641.
8. Yusuf D, Butland SL, Swanson MI, Bolotin E, Ticoll A, Cheung WA, Zhang XY, Dickman CT, Fulton DL, Lim JS, et al. The transcription factor encyclopedia. Genome Biol. 2012; 13:R24.
